# Measuring the morphological characteristics of thoracolumbar fascia in ultrasound images: an inter-rater reliability study

**DOI:** 10.1186/s12891-018-2088-5

**Published:** 2018-06-01

**Authors:** Kyra De Coninck, Karen Hambly, John W. Dickinson, Louis Passfield

**Affiliations:** School of Sport and Exercise Sciences, University of Kent, Medway Building, Chatham Maritime, Chatham, Kent, ME4 4AG UK

**Keywords:** Inter-observer reliability, Thoracolumbar fascia, Ultrasound imaging

## Abstract

**Background:**

Chronic lower back pain is still regarded as a poorly understood multifactorial condition. Recently, the thoracolumbar fascia complex has been found to be a contributing factor. Ultrasound imaging has shown that people with chronic lower back pain demonstrate both a significant decrease in shear strain, and a 25% increase in thickness of the thoracolumbar fascia. There is sparse data on whether medical practitioners agree on the level of disorganisation in ultrasound images of thoracolumbar fascia. The purpose of this study was to establish inter-rater reliability of the ranking of architectural disorganisation of thoracolumbar fascia on a scale from ‘very disorganised’ to ‘very organised’.

**Methods:**

An exploratory analysis was performed using a fully crossed design of inter-rater reliability. Thirty observers were recruited, consisting of 21 medical doctors, 7 physiotherapists and 2 radiologists, with an average of 13.03 ± 9.6 years of clinical experience. All 30 observers independently rated the architectural disorganisation of the thoracolumbar fascia in 30 ultrasound scans, on a Likert-type scale with rankings from 1 = very disorganised to 10 = very organised. Internal consistency was assessed using Cronbach’s alpha. Krippendorff’s alpha was used to calculate the overall inter-rater reliability.

**Results:**

The Krippendorf’s alpha was .61, indicating a modest degree of agreement between observers on the different morphologies of thoracolumbar fascia.The Cronbach’s alpha (0.98), indicated that there was a high degree of consistency between observers. Experience in ultrasound image analysis did not affect constancy between observers (Cronbach’s range between experienced and inexperienced raters: 0.95 and 0.96 respectively).

**Conclusions:**

Medical practitioners agree on morphological features such as levels of organisation and disorganisation in ultrasound images of thoracolumbar fascia, regardless of experience. Further analysis by an expert panel is required to develop specific classification criteria for thoracolumbar fascia.

## Background

A growing body of evidence supports the notion that the thoracolumbar fascia, an anatomical structure consisting of layers of dense connective tissue in the lumbar area of the trunk, is clinically important in people with chronic lower back pain [1–8]. The thoracolumbar fascia has been shown to play an important role in force transmission between lower limbs and trunk in both ex-vivo cadaver studies [9, 10] and in-vivo research during walking [11, 12]. Subcutaneous fascial bands have been found to mechanically link the skin, subcutaneous layers and deeper muscles. The differences in morphological characteristics of subcutaneous fascial planes may reflect how mechanical forces are distributed across various tissues [13]. However, what is not clear, is whether medical practitioners are able to agree on these different morphological features in ultrasound images of thoracolumbar fascia.

The architecture of the thoracolumbar fascia is complex, it consists of layers of dense collagenous connective tissue, interspersed with loose connective tissue which allows the dense layers to slide and hence play a role in trunk mobility. The thoracolumbar fascia is continuous with the aponeuroses of major trunk muscles which are instrumental in movement and vertebral control [8, 9]. It has been hypothesised that fibrosis, densification and thickening in the thoracolumbar fascia may be the result of an inflammatory response or soft tissue injury [1, 14–17]. For instance, a recent animal study demonstrated that an induced soft tissue injury in the lumbar region, when combined with movement restriction, lead to fibrosis, and significant thickening of thoracolumbar fascia [18]. An earlier pioneering ultrasound based human study concluded that the thoracolumbar fascia in people with chronic lower back pain demonstrated 25% greater thickness compared to a matched control group [4]. A follow-up investigation found that thoracolumbar fascia shear strain during passive trunk flexion, was reduced in people with chronic lower back pain by 56% [19]. In both aforementioned studies, Langevin’s research team found significant differences not only in fascial thickness and echogenicity, but also in disorganisation of the architecture of the connective tissues of people with chronic lower back pain. Even though the clinical relevance of fascial tissues has been established [20], to date no classification of thoracolumbar fascia has been developed. In order to develop a classification system, a level of inter-observer reliability of the different types of architecture of thoracolumbar fascia needs to be established.

The aim of this study was to determine the inter-rater reliability for the rating of morphological characteristics of thoracolumbar fascia in ultrasound images, on Likert-type scale, by a range of clinicians.

## Methods

### Participants

The study was approved by the University of Kent’s Ethics Committee and conducted in compliance with the Helsinki Declaration. Informed consent was obtained from all participants.

The inclusion criteria for participants were: medical professionals in the orthopaedic, sports medicine or sport rehabilitation field, with or without ultrasound experience or training. Twenty raters were recruited at a European Sports Medicine symposium to rate the scans independently, in a group setting. Subsequently, a further 10 participants were recruited through opportunistic sampling (see Table 1 for characteristics). This group viewed the scans individually on a standard size desktop PC computer (screen size 50 × 28 cm). These participants received the same presentation on thoracolumbar fascia. All scans were anonymised and displayed in randomised order. All participants viewed all 30 scans. Participants were asked about clinical training, years of clinical experience, musculoskeletal ultrasound training, and frequency of ultrasound image usage for diagnostic purposes in clinical practice.

**Table 1:** Characteristics of raters

Clinical training	*N* = 30
MD	21 (70%)
Physiotherapists	7 (23%)
Radiologists	2 (6%)
Years of clinical experience	13.03 (± SD 9.6)
USI training & experience	*N* = 30
Trained & experienced	12 (40%)
Untrained & unexperienced	17 (57%)
not known	1 (3%)
Frequency of USI usage	*n* = 12 (40%)
daily	4 (33%)
weekly	4 (33%)
monthly	4 (33%)

USI = ultrasound imaging

### Ultrasound image data acquisition

**Fig. 1:**
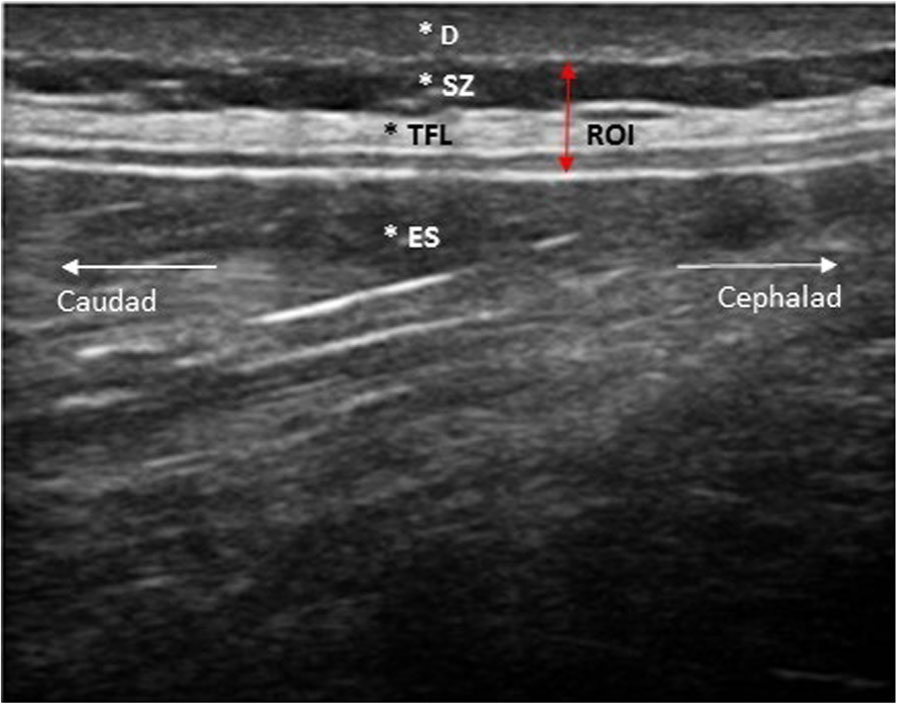
Anatomical orientation and delineation of the zones rated. *D = dermins. *SZ = subcuteanouz zone. *TFL = thoracolumbar fascia. *ES = erector spinae. ROI = region of interest, zones rated

Images were taken at the intervertebral level 2–3, as fascial planes are the most parallel to the skin at this level [4]. The interspinous ligament between lumbar vertebrae 2 and 3, and the superficial border of posterior paraspinal muscles were identified using a validated protocol [21]. One focal region was set as close as possible to the thoracolumbar complex. Bi-lateral parasagittal (longitudinal) images were taken 2 cm lateral of the intervertebral disc space between lumbar vertebrae 2 and 3. The image acquisition was based on a validated protocol [4]. All images presented to raters were obtained using uniform settings, a frequency of 18 MHz was used, with a depth of 3 cm, which allow optimum image quality for subcutaneous structures [22]. See Fig.1 for example of ultrasound image and anatomical orientation.

Each ultrasound image was obtained using B-Mode imaging, with a MyLabGold25 semi-portable ultrasound scanner (Easote, Rimini, Italy). A 4 cm, 18 MHz linear array transducer (Easote LA435) was used for all images.

### Selection of ultrasound images for reliability study

**Fig. 2:**
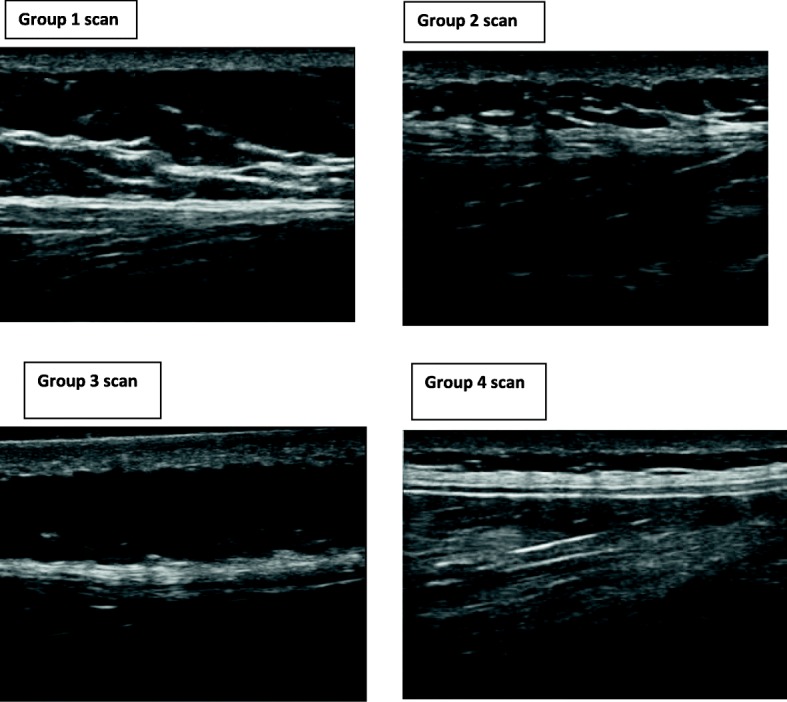
A range of different thoracolumbar fascia morphologies. Sub-groups of different TLF morphologies. Group 1 = example of ‘very disorganised’, Group 2 = ‘somewhat disorganised’ Group 3 = ‘somewhat organised’, Group 4 = ‘very organised’ . The sub-grouping was based on the median scores for each scan

Initially, a single investigator selected 40 scans from a data-base of 308 bi-lateral scans of 154 male and female subjects with and without lower back pain from a larger prior study. A focus group then viewed the 40 images and selected 30 scans. Both the individual investigator and the focus group were instructed to select scans which, in their opinion, represented both ‘organised’ perimuscular fascia and ‘disorganised’ perimuscular fascia, with a range in between. ‘Organised’ was defined as ‘being able to draw a rectangular box’ around the hyperechoic zone, ‘disorganised’ was described as ‘not being able to draw a rectangular box’ around the hyperechoic zone. All raters were blind to any pathology or background information related to the scans. These 30 scans were deemed to represent the range of morphologies from very disorganised to very organised and a range of scans in between (Fig. 2).

### Inter-observer reliability rating protocol

In inter-observer reliability studies, it is vital that raters apply coding to data they understand [23]. For this reason, a 20 mins presentation about the thoracolumbar fascia was delivered, this facilitated anatomical orientation and exposed the participants to a representative range of ultrasound images prior to rating. Participants were not given examples of actual ratings, only of the range of images they would be rating, to avoid bias. (See Fig. 1 for anatomical orientation and region of interest). Scans were projected on a standard sized screen (133 × 100 cm).

Table 1 shows that 57% had no training or experience in ultrasound imaging, 40% had experience ranging from monthly to daily evaluations of ultrasound imaging, 1 participant did not respond to this question, no observers had experience in evaluating ultrasound images of thoracolumbar fascia.

Participants were instructed to rank the region of interest (ROI in Fig. 1) which included the thoracolumbar fascia (* thoracolumbar fascia in Fig. 1) and the subcutaneous zone (*SZ in Fig. 1) on a Likert-type scale. A Likert scale with rating points from 1 to 10 was used, point 1 was labelled as ‘very disorganised’ and point 10 as ‘very organised’, the intermediate points were numbered but remained unlabelled. Participants were familiarised to the definition of thoracolumbar fascia organisation and disorganisation. For instance, ‘very organised’ was defined as ‘to be able to draw a rectangular shaped box around the hyperechoic area of thoracolumbar fascia’ (see Fig. 1).

Participants viewed scans sequentially in a time frame of 30 s to 1 min. They were able to modify responses, request to re-assess a scan, and make written comments about their decisions. Participants could not discuss ratings with each other, in order to avoid bias. All responses were anonymised prior to analysis.

### Statistical analysis

Inter-rater reliability was assessed from the total raw scores of all 899 decisions, and the raw scores divided into 4 sub-groups using Cronbach’s alpha, to assess internal consistency among observers [24, 25]. The Cronbach’s alpha coefficient was calculated using SPSS (version 21) statistical software. Standard error of measurement (SEM) was calculated as the square root of error variance in accordance with de Vet’s guidelines [26]. The Krippendorff’s alpha for ordinal measures was used to assess inter-observer agreement [23, 27] and was calculated using a custom-designed online calculator [28]. As Likert scales are an ordinal measurement, the median and interquartile range for the total of scans was calculated, as well as for each scan individually [29, 30].

Participant ratings of scans were categorised into four groups [30–32]. Group 1 (very disorganised) consisted of all scans with a median rating of 1 to 3. Group 2 (somewhat disorganised) consisted of all median ratings from 4 to 5. Group 3 (somewhat organised) consisted of all median ratings from 6 to 7. Group 4 (very organised) consisted of all median ratings from 8 to 10 (Fig. 2). The Cronbach’s alpha and Krippendorf’s alpha were calculated using the original raw scores from individual raters for each scan.

## Results

### Results of descriptive analysis

**Fig. 3:**
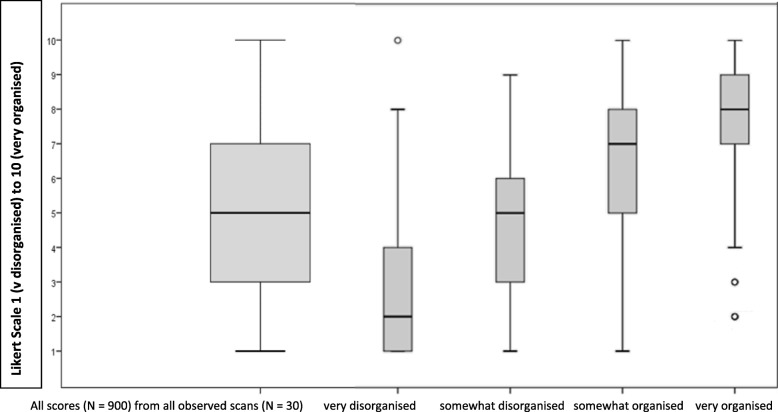
Box-plots of all ratings, and ratings for each sub-group. Boxplots for total scores of the ratings (899 decisions) and ratings for each sub-group. Central tendency is the median, distribution is the interquartile range

The median (m = 5) and interquartile range (IQR = 4) of the total ratings were calculated (range = 1–10), as well as for each group (Table 2 and Fig. 3).

**Table 2:** Inter-rater reliability scores for all data and sub-groups

Group	Decisions (%)	Median (IQR)	Cronbach’s alpha	Landis and Koch criteria [33]	SEM
All data	899	5 (4)	.98	excellent	0.10
Group 1	300 (32.8%)	2 (3)	.70	excellent	0.40
Group 2	209 (22.6%)	5 (3)	.68	good	0.17
Group 3	150 (20.3%)	7 (3)	.47	moderate	0.56
Group 4	240 (24.2%)	8 (2)	.56	moderate	0.50

*SEM* standard error of measurement, *Group 1* very disorganised, *Group 2* somewhat disorganised, *Group 3* somewhat organised, *Group 4* very organised

### Results of inter-rater reliability analysis

All participants assessed all scans, except one participant who did not complete one rating. The Cronbach’s alpha was 0.98, which is considered excellent according to the Landis and Koch criteria [33]. Observers without ultrasound imaging experience scored a Cronbach’s alpha = 0.96, observers with ultrasound imaging experience scored a Cronbach’s alpha = 0.95, both in the excellent range. Scores between 4 sub-groups are reported in Table 2. The Krippendorff’s alpha for ordinal measures was .61, with an error variance of 0.63, indicating a modest degree of agreement.

## Discussion

In this study we found that medical practitioners agree on different morphological features in ultrasound images of thoracolumbar fascia such as levels of organisation and disorganisation. This agreement is independent of experience in ultrasound image rating. We found that the knowledge gap between musculoskeletal (MSK)-trained radiologists, MSK-trained medical doctors and physiotherapists on the one hand, and clinicians untrained and inexperienced in MSK ultrasound, did not affect the inter-observer agreement.

It is important to establish internal consistency before images can be used for research or clinical evaluation to ensure validity [24]. The measurement error was smaller in both groups of disorganised scans, and higher in the more organised groups. This could be an indication that it may be easier to interpret disorganisation or irregular shapes rather than organisation or regular shapes. The modest Krippendorf’s alpha for the ratings suggests that a minimal amount of measurement error was introduced by the independent observers, and therefore statistical power for subsequent analyses is not substantially reduced.

In this cohort, the differences in ultrasound experience do not appear to impact on consistency. We did not observe any raters who systematically under- or over-rated the images. Novice raters have demonstrated good to excellent reliability in measuring abdominal and lumbar muscle thickness obtained by ultrasound scans [34, 35]. However, a straightforward comparison between quantitative measures of lumbar and abdominal muscle tissue, commonly found in the literature on rehabilitation of lower back pain, and this study’s qualitative ratings of subcutaneous connective tissue requires caution. Substantial observer variability can occur, even at the expert level of image interpretation [36]. Interestingly, in this study, experienced radiologists agreed with the interpretation of clinicians relatively inexperienced in the reading of ultrasound images. The American College of Radiology Imaging Network (ACRIN) has highlighted that in order to improve the research in interpretation of medical images, observers in reliability studies should ideally reflect a broad range of experience to provide a sufficient level of generalisability [37].

In multi-reader medical image interpretation, the phenomenon of ‘groupthink’, has been identified, where the opinion of novice raters might be influenced by senior or experienced raters [36]. In order to avoid a situation of potential pseudo-consensus, all raters viewed the scans independently without discussing decisions with each other.

This study has a number of limitations. First, it involved a small cohort size of both observers and scans. The results are encouraging and should be validated in a larger cohort [37]. Secondly, the study relied on static ultrasound images. Future studies may consider functional and dynamic measurements. Finally, we did not determine the frequency in which raters interpret the same image differently. This needs to be taken into account for future studies.

## Conclusion

Medical practitioners agree on morphological features such as levels of organisation and disorganisation in ultrasound images of thoracolumbar fascia, regardless of experience. These findings will be useful for the establishment of a clinical diagnostic scale and the further development of using ultrasound as a decision-making tool for researchers and clinicians.
